# Mitigating inequalities in community care needs of older adults with dementia: a qualitative case study of an integrated model of community care operated under the proportionate universalism principle

**DOI:** 10.1186/s12875-022-01855-z

**Published:** 2022-09-21

**Authors:** Siu-Ming Chan, Gary Ka-Ki Chung, Michelle Ho-Wing Kwan, Jean Woo

**Affiliations:** 1 CUHK Institute of Health Equity, The Chinese University of Hong Kong, Hong Kong SAR, China; 2 Department of Social and Behavioural Sciences, City University of Hong Kong, Kowloon Tong, Hong Kong SAR, China; 3 CUHK Institute of Ageing, The Chinese University of Hong Kong, Hong Kong SAR, China; 4 Department of Medicine & Therapeutics, Faculty of Medicine, The Chinese University of Hong Kong, Hong Kong SAR, China

**Keywords:** Integrated care, Voucher, Equity, Public-private partnership, Dementia, Caregiver

## Abstract

**Background:**

Population ageing and community care on older adults, as well as the marked social inequalities in health, have received growing concern by the government and the community. This study evaluated the medico-social integrated day care model of the Cadenza Hub for older adults with dementia. We also examined whether services subsidized by the publicly funded graded financial support of the Community Care Service Voucher for the Elderly (CCSV) could mitigate social inequalities in community care needs, from the perspective of the caregivers.

**Methods:**

In this qualitative case study, we adopted purposeful sampling strategy to recruit 14 caregivers of active day care service users with dementia, with different socioeconomic background and duration of service use, for face-to-face semi-structured in-depth interviews between June and August 2021. The transcribed data were closely read to capture key themes using thematic analyses.

**Results:**

Caregivers faced tremendous caregiving burden in the absence of community care support and struggled in choosing care services. Most informants benefited from the day care service, whereas the financial support of CCSV was crucial to ensure equitable access to community care. Non-governmental organizations and social workers were the key to bridging the information gap.

**Conclusion:**

The integrated day care of the Cadenza Hub appeared to have addressed the unmet needs of older adults with dementia and their caregivers, including the socioeconomically disadvantaged with the CCSV support. The community care service delivery model might be applicable to address other health inequalities problems.

## Introduction

Population ageing is a challenging societal and public health issue, especially in developed Asian regions like Hong Kong with the longest life expectancy across the globe [1, 2]. Over the recent decades, promoting healthy ageing in the community has gained increasing research and policy attention, over and above the conventional focus on longevity and disease prevention [3]. As highlighted by the World report on ageing and health of the World Health Organization, the health status of older adults may not be measured solely by the occurrence of chronic diseases, disabilities, and life expectancy, but also by alternative measures of healthy ageing which emphasizes both physical and cognitive functions [4].

Nonetheless, there is ample evidence on social inequalities in health among older people in Hong Kong, where a social gradient exists in declines in cognitive and physical function, in depression, in avoidable hospital admissions, and in healthy life expectancy [5–12]. Geographic variations in health outcomes are also commonly observed across districts, which may have been shaped by both the physical and social environment [13–15]. A significant contributor to these inequalities is ageism, a prominent phenomenon in Hong Kong [16] which results in neglect of policies directed to the design and provision of services targeted to needs, slow development of a community continuing care system that is fit-for-purpose [17], as well as inadequate training of health and social care professionals at all levels of care for older adults. In other words, such a stigma affects the availability of community services and workforce to support older adults in need; thereby creating extensive unmet community care needs [17] and increasing caregiving stress [18, 19], especially among the socioeconomically disadvantaged.

In many developed economies in Asia which have been experiencing population ageing, there is a move towards medico-social integration care models in the community that covers preventive as well as needs-based long-term care, financed by long-term care insurance [2]. Nonetheless, in Hong Kong, such services are either rudimentary, unaffordable, or of low quality and not targeted towards preserving or optimizing function. As the primary care services are largely private with substantial out-of-pocket charges, for many older adults who cannot afford this, the first point of contact of care is the Accident and Emergency Department of public hospitals since this service is free or at low cost. In the absence of long-term care insurance or mandatory healthcare insurance, the Hong Kong government has recently launched a means-tested Pilot Scheme on Community Care Service Voucher for the Elderly (CCSV) with different levels of co-payment [20]. Following the “affordable users pay” principle, older adults are required to pay at the rate of 5, 8, 12, 16, 25%, or 40% of the community care service package value of the voucher depending on family household income but not asset, while the Hong Kong government pay for the rest of package value [20]. Thus, the CCSV scheme provides different levels of safety net for community-dwelling older adults in need across the social ladder.

With the graded financial support by the Government, the existing inequalities in community care needs in Hong Kong could possibly be addressed if effective community care services are also in place. One exemplary community service is the Cadenza Hub, a novel medio-social integrated community care model for older adults in Hong Kong. It is a community service project initiated and funded by The Hong Kong Jockey Club Charities Trust in 2009 in response to the challenges of population aging [21, 22], which aims at promoting an age-friendly community and improving the quality of life of older adults as well as their caregivers in Hong Kong [23]. Currently, the day care service of the Cadenza Hub is largely targeted at older adults with dementia. Although service utilization in the Cadenza Hub, like most private services, tended to be pro-rich due to its inherent self-financed nature, the recent launch of the above-mentioned CCSV scheme could make a profound change in service access. Hence, given the potential equity impact of this public-private partnership in providing medico-social integrated day care in the Cadenza Hub under the CCSV support, the present study aimed to explore the views of the caregivers on (i) the experience of service use in the Hub and (ii) whether this service model is effective in mitigating social inequalities in community care needs of older adults with dementia and their own caregiving burden.

### Research methods

#### Study population

This qualitative study adopted a case study research design to evaluate the subjective experience of caregivers of older adults with dementia who received service in the Cadenza Hub. Purposeful sampling method was adopted to recruit caregivers or close relatives of active users of the Cadenza Hub via referrals by the Hub manager. This sampling approach enabled an in-depth investigation into the day care service experience from the perspective of their caregivers, as well as the subjective feeling and views of the caregivers themselves. To maximize the variation of sample, 14 caregivers with varying socioeconomic backgrounds, and duration of day care service utilization were recruited via phone calls for individual interviews (Table 1). The sample size was determined by data saturation when no new themes from participants’ experiences emerged.

**Table 1:** Basic characteristics of participants

Case no.	Sex	Age range	Education level	CCSV level	Dementia level of user	Year of service use	Living with user	Hiring a maid
1	M	50–59	Senior high school	II	Mild	1–2	No	No
2	M	60–69	Junior high school	II	Moderate	3–4	Yes	No
3	F	50–59	Junior high school	I	Severe	> 4	Yes	No
4	F	60–69	Undergraduate	II	Mild	1–2	Yes	No
5	F	40–49	Undergraduate	III	Mild	> 4	No	No
6	F	50–59	Senior high school	IV	Severe	> 4	No	Yes
7	M	80–89	Primary school	I	Moderate	1–2	Yes	No
8	F	60–69	Senior high school	II	Severe	2–3	Yes	No
9	M	60–69	Undergraduate	IV	Moderate	1–2	No	Yes
10	F	70–79	Junior high school	II	Mild	< 1	Yes	No
11	F	70–79	Primary school	III	Mild	1–2	Yes	No
12	M	70–79	Junior high school	I	Moderate	3–4	Yes	No
13	F	60–69	Primary school	II	Severe	< 1	Yes	No
14	F	50–59	Senior high school	II	Moderate	2–3	Yes	No

#### Data collection

Participants were interviewed by two researchers trained with qualitative interview skills (i.e., a male postdoctoral fellow – S.M.C., PhD and a female research assistant – M.H.W.K., BSc) between June and August 2021. There was no prior relationship and knowledge between the interviewers and the participants. A semi-structured interview guide was developed with reference to the existing literature on community care, gerontology, and social services. Participants were also invited to openly share their views on the service of the Cadenza Hub and community care in Hong Kong. Examples of questions included: *(i) What are the major difficulties you faced as a caregiver? (ii) What do you think about the difference between community day care and old age home? What are the considerations behind your decision? (iii) How long did you use the Cadenza Day Care service? Can you share with me your daily experience in the Hub? (iv) What are your opinions on the Cadenza Day Care service? (using probes on types of services, case management, health promotion, service hours,* etc. *when appropriate) (v) What do you think about the service charges in the Cadenza Hub? (vi) What do you think about the amount and application of the government voucher scheme (*i.e.*, CCSV) in terms of facilitating access to day care service? (vii) From the perspective of a caregiver, what kind of additional help and support do you expect?* All the participants filled a fact sheet about their socioeconomic position and family background. The interviews were conducted in Cantonese for around 45 to 60 minutes each in the Cadenza Hub, which were audio-recorded with the consents of participants.

#### Data analysis

The interview data were transcribed verbatim into Chinese texts by trained student helpers and checked by the interviewers before analysis. Thematic analysis strategy was applied following the suggestions of Braun and Clarke [24]. We followed the steps by having S.M.C., M.H.W.K., and G.K.K.C. (another postdoctoral fellow), to first familiarize themselves with the data by independently reviewing each transcript in detail, and then generated initial codes and derived preliminary themes from the data. To ensure the scientific rigor and trustworthiness of this study, we invited other researchers to review the coding and refine the derived themes, which helped confirm whether the coding and themes are understandable to readers [25, 26]. Moreover, the data transcripts were reviewed several times with considerations of the corresponding field notes taken during the interviews to enhance the credibility of our thematic analysis [27]. The quoted transcripts and formulated coding were translated into English by bilingual researchers. Back translation was also applied to ensure the accuracy of the quotes. The data analysis put special attention to the experience of caregiving, use of day care service, and opinions on the community care. Excel spreadsheet was used for manual coding and management of the identified quotes.

## Results

### Background of participants

Among the 14 participants, all of them are caregivers of the Cadenza Hub users diagnosed with mild, moderate, or severe levels of dementia. Nine of them were female and five were male. The duration of day care service use of their care recipients ranged from 2 months to more than 6 years. All of them used CCSV with varying co-payment levels ranging from level I to level IV. For further background information of the participants please refer to Table 1.

### Key findings

Five key themes emerged in this study. The first two themes were about the difficulties and struggles faced by the caregivers and service users under the current community care system in Hong Kong, which included (i) Tremendous caregiving burden in the absence of community care support and (ii) Struggle in choosing care services. The next two themes focused on how the Cadenza Hub service and its service delivery model could work synergistically to mitigate the existing social inequalities in community care needs under the CCSV scheme, which included (iii) Benefits of the day care service and (iv) Financial support to ensure equitable access to community care. Finally, the last theme illustrated how efforts in the community sector could enhance health equity by matching appropriate community care services to those in need, i.e., (v) The role of NGO and social worker in bridging the information gap. Details of the five themes are elaborated as follows:

### Theme 1: tremendous caregiving burden in the absence of community care support

Taking care of older adults with dementia was a huge challenge for caregivers. Many caregivers expressed their sorrow and frustration during the interviews, as they did not have any professional knowledge of dementia and had no training on caregiving before using the Cadenza Hub service. With limited experience, caregivers felt devastated, including those whose care recipients were still at the early stage of dementia but not eligible for public service referral due to the absence of official diagnosis of dementia.

‘When I started to be a caregiver, I was incompetent, I didn’t know how to care for my mother. I felt devastated. One time, my mum was at the hospital and my brother got a stroke. My brother had gone crazy, both of them. I was so helpless at that time … No one can help me out. I had to take care of everything by myself, for both of them… My relatives and friends do not live nearby, so it is hard to ask them to come and help without a strong reason. Fortunately, here (Cadenza Hub) offered a dementia assessment for my mum and found the causes of her abnormal behaviour, then I realised it is because of dementia.’ (Case 3, female, 53)

I was devastated at that time (after my mother was discharged from the hospital). I had no idea what to do when we were home. She could not take care of herself. We didn’t let her sleep in her room, as it is easier to look after her when we placed her in the living room … The only thing we can do now is to take care of her, not any other things. For example, I rarely hang out with my friends, luckily my friends are considerate and willing to accommodate my needs when scheduling the time to meet. (Case 8, female, 62)

Many of the informants were at a loss when taking up the caregiving role all of a sudden, and needed to put a lot of time and effort on caregiving by giving up their own social life. They were worried about the family members with dementia days and nights, given the deterioration of ability to self-care. Without the community care service, the caring burden would have been even heavier and have more seriously disrupted their daily lives. Community care services providers, such as the Cadenza Hub, provided dementia assessments and supporting services to help them out considerately. With the support, they gained a better understanding on the behavioural and psychological symptoms of their family member with dementia, and had a clearer idea and expectation on their caregiving role.

### Theme 2: struggle in choosing care services

Choosing appropriate care services for older adults was a common challenge for all caregivers. While possible options include applying for residential care homes, employing foreign domestic helpers, visiting community centres, or using community day care services, caregivers tend to prefer community day care services despite a greater caregiving role, and put residential care homes as their last resort due to the less desirable environment.

The caregivers wish their family members with dementia could join professional training to maintain physical health and prevent memory loss. However, they tended to believe that most aged care homes in Hong Kong do not have the capability to carry out professional training for the aged residents.

Because my brother is living in the aged care, I always witness the problems of the aged care services. I would rather take up more by myself but to avoid sending my mother to the aged care. At that time, my mother was still able to walk, or using a wheelchair… I wish to keep her out of the aged care, so that she can (be free to) do exercise in the daytime. I mean she can stay here and receive training to prevent memory loss. At the aged care, the care workers don’t care about it at all. They just let the residents sleep all day. So, that’s the reason why I prefer my mother staying here (Cadenza Hub) to living at the aged care, so she can practice how to take care of herself. (Case 3, female, 53)

Also, participants generally agreed that the environment as well as the services in the Cadenza Hub were much better than those of residential care homes and could not be made possible by simply hiring a foreign domestic helper. The centre provides cognitive and muscle training, therapeutic services, and social activities for the older adults with dementia during day time, and the members can return home in the evening. The service users only need to get used to the schedule at the centre and remain the rest of the living routine at home.

I had visited a lot of elderly centres, including service centres. The Cadenza Hub is really clean, tidy, and new. I think the Cadenza Hub belongs to a first-class facility, probably. It is unbeatable. Whenever you go to other centres, you will find a great difference. (Case 1, male, 57)

Considering the current situation, I prefer the day care centre. It is because my husband and I can still take care of my mother. The day care service is a better choice for older adults as they are more familiar with the environment at home. Just like children would be reluctant to go and adapt to a new environment. (Case 14, female, 57)

My mum loves to come here (Cadenza Hub) because she didn’t get to go to school when she was young. So, she is so glad to join the social activities here. Normally, she doesn’t like to do exercise. After coming to the hub, she keeps doing exercise regularly. She became more energetic and healthier than before, and developed a regular lifestyle. Now, her condition is very well, I noticed that the oedema on her legs has improved after doing the aroma massage therapy. (Case 4, female, 64)

Overall, caregivers initially struggled in choosing among various types of care services. Nevertheless, most of them preferred community day care services rather than elderly homes where the living environment and services were not satisfactory. They were seeking services which are helpful to their family members with dementia to maintain both physical and mental health. Moreover, the professional training and diverse services in the Cadenza Hub cannot be replaced by the aid of domestic helpers, suggesting the niche of the community day care service in the Hub.

### Theme 3: benefits of the day care service

The significant improvement of physical ability and mental health of service users explained why they prefer to stay in the Cadenza Hub. Most of the caregivers initially felt hopeless when they knew their parents or relatives suffered from dementia, but the improvement of service users in the Hub gave them hope again. As the informants reflected, their family members showed significant improvements in social skills, self-care abilities, and emotional control.

He has made a lot of improvements over this year. In the beginning, he had no contact with others. No eye contact, nothing. He won’t care about anything when he got into the lift. Then he got better, he has started to say hi to people and took the initiative to greet people. And now, he even chats with others. The centre (Cadenza Hub) has made a lot of positive changes in his personality… The centre has changed him a lot by showing a great improvement in changing clothes and cleanliness. His social skill is getting better and this emotion is also well stable, less likely to lose his temper. (Case 1, male, 57)

(What do you find helpful in the one-on-one exercise class?) We have learnt a set of skills, such as when she gets up to sit on a wheelchair… she has to hold on to the table to get up, then sits on the wheelchair behind her. We didn’t know all this before taking the class. (Case 8, female, 62)

The day care centre helps a lot. We have pushed back the date of admitting to the elderly home. The Cadenza Hub is near my home, we live upstairs. It provides cognitive training which I don’t know how to train her myself. The services provided at the centre are more professional than what family can do, it should be helpful… (Case 12, male, 74)

The service provided by Cadenza Hub not only enhanced the well-being and promotes healthy ageing of the older adults, but also benefited the caregivers. As most caregivers bear huge pressure in taking care of their parents or relatives, the day care service gave them appropriate support and free time for their own commitment. Most informants agreed that day care service allowed caregivers to have more leisure time, enjoy their social life with friends and families, and work for a living. They felt disburdened with the support from the Cadenza Hub.

(Does the service provided by Cadenza Hub help in reducing your stress?) Yes, of course. Cadenza Hub takes care of her for a few hours, she can do some exercise, chatting with others… It would be nice to always be with her, but I have to work and sustain a living. There is a benefit to go to work, which I don’t need to stick with her all day long. I don’t think being with her is something I can’t accept. I’d like to stay with her, but I have to go to work. It is absolutely helpful. (Case 2, male, 60)

(Any changes after you joined the Cadenza Hub day care centre?) There is more time for me to relax. I can go out for my activities, or to relax for a few hours. And here (Cadenza Hub) has taught me a lot. For example, when my mother is unable to take a shower on her own, the staff would teach me how to hold my mother at the shower… with opening wounds, the staff would take a look at it and teach me how to manage it. I am impressed with the details of caring at the centre. The staff are always welcome for questions. (Case 3, female, 53)

Most of the caregivers appreciated the services delivered by the community care centre, especially those who need to work in the day time. The community day care service and the responsible staff in the Cadenza Hub not only allowed the caregivers to work and enjoy social life without much worry, but also offered both psychological and social support by equipping them with better caregiving skills to facilitate dementia care at home and coping skills to manage their own well-being when feeling overwhelmed.

### Theme 4: financial support to ensure equitable access to community care

Most existing community care services are private and self-financed by nature with substantial out-of-pocket payment. Taking the Cadenza Hub as an example, the regular fee is up to $400–500 Hong Kong dollar (equivalent to around US$50–65) per day. Therefore, many caregivers, especially the socioeconomically disadvantaged, expressed that they would not have been able to afford the day care service in the absence of the government financial support via CCSV.

‘There is no doubt that the social service vouchers (CCSV) have helped me a lot. It is better to receive financial aid from the government. If not, we won't be able to join the Cadenza Hub or the day care centre. How can someone not receiving the CCSV join the Cadenza Hub? It is affordable when it is subsidized with the service vouchers… We need to pay 5% of the fee only, around $300 HKD per month… Without the vouchers, it would be $500 HKD per day. If paying the total fee out of my pocket, I won’t be willing to spend that much, unless I am wealthy.’ (Case 12, male, 74)

‘We got the service vouchers (CCSV) unexpectedly, which can save some money for her to join the aroma massage therapy and one-on-one exercise in Cadenza Hub. Since my mum’s health condition has improved, less money is being spent on other medical care.’ (Case 4, female, 64)

‘The service voucher (CCSV) helps a lot financially, it makes a big difference, the regular fee is more than ten-thousand dollars!… If I pay the regular price, I have to pay more than ten-thousand dollars a month, which is really expensive. Without the voucher, I will never join the Cadenza Hub. It is more than $400 HKD per day.’ (Case 13, female, 62)

Quality community care service may not necessarily be affordable to the families in need. Many caregivers struggled in the trade-off between the cost and quality of service. Also, certain professional training and therapeutic services may even require extra fees, deterring the low-income families from service use. With the financial support provided by the government, their choices in access to professional and quality services were significantly improved. Since the CCSV scheme provided graded subsidy based on the principle of proportionate universalism, families with lower income were eligible for a greater amount of subsidy; thereby promoting a more equitable access to services in the community.

### Theme 5: the role of NGO and social worker in bridging the information gap

Social workers and NGOs played an important role in bridging the gap between the clients in need and the existing community services. Many caregivers had no ideas about community care services before getting in touch with social workers, whereas the advertisements of community services were often scattered and hence can hardly reach the cases in need. As the informants reflected, they have difficulty in mapping the existing services themselves, and agreed that social workers and NGOs are the most effective channel for linking their needs and community care services providers.

We have seen many advertisements at the estate about the community services for the elderly. However, you won’t know what it is if you never come close to check. The elderly won’t notice there are services for them. They also don’t get on to the internet. (Case 5, female, 45)

(How do you know about Cadenza Hub?) It is mainly recommended by the medical social worker. The social worker has introduced what sort of services are provided at the hub to me, which I find it suitable for my mother-in-law to join. Therefore, we have joined since then. (Case 6, female, 53)

In addition, social workers provided a channel for the caregivers to get access to information about the existing social welfare and administrative support on applying for the relevant schemes.

When I was applying the service vouchers for my sister, the medical social worker helped me out. She did the assessment of our case, and we are eligible to get it, then she brought the vouchers to us. This social worker has helped me a lot. (Case 9, male, 62)

Most of the service users applied for the service mainly via referral by social workers or field workers in the community centres. This theme highlights that, while the provision of community care services is crucial, it is also important to have social workers and NGOs to line up the families in need with the existing services and welfare schemes in order to facilitate a more equitable access to quality services in the community.

## Discussion

### Summary of findings

The novel and self-financed medico-social integrated day care model of the Cadenza Hub appeared to have addressed the unmet needs of older adults with dementia and their caregivers, including those of a relatively lower socioeconomic position with the support of the publicly funded CCSV scheme. Substantial improvement on the self-care, functionality, social skills, and psychosocial well-being of older adult users across different socioeconomic background has been observed, in addition to stress relief among caregivers. Despite limitations on service referral and delivery, the day care service has the potential to fill the gaps of existing services and thus retain older adults with dementia in the community as long as possible.

### Addressing the unmet community care needs via the day care model

The day care model of the Cadenza Hub tackles the unmet community care needs through a series of inter-related support including a comprehensive geriatric assessment followed by multidisciplinary and person-centred management for both dementia-specific illnesses and more general health and psychosocial wellbeing, technological assistance and training for older adult users, proactive support for caregivers, as well as a comfortable setting that facilitates social interactions with other older adults with dementia and their caregivers [21, 22]. The design of the day care model echoes with the 8 Pillars Model of Community Support developed by Alzheimer Scotland which emphasizes resilience building by providing the best possible health and social support to people with dementia to live in their local community [28]. A recent systematic review also supported the concrete benefits of organizational-level patient and family-centred care on the quality of life of older adults with dementia [29]. Hence, the day care model goes beyond rehabilitative programmes but primarily optimizes health and function of older adult users in terms of the physical, psychological, functional, nutritional, and social domains, and provides caregiving support to facilitate continuity care at home and in the community [23]. As community care is often a priority over hospital and residential care, the day care service supports older adults with dementia who have already been discharged from hospitals but are still in need of care to stay in the community, so as to avoid frequent hospital readmissions or resorting to residential care home services where many users suffer from a loss of dignity while their caregivers may also feel guilty under the traditional culture of filial piety [30]. Moreover, the day care centre provided skill training and social activities which cannot hardly substituted by hiring a foreign domestic helper. Therefore, it is believed that the one-stop day care service could address the fragmentation between the healthcare and residential care sectors, enabling older adults with dementia to stay in and re-adapt to the community for healthy ageing.

### Mitigating the inequalities in community care needs with the CCSV support

In addition to the direct health and social benefits to older adult users and their caregivers, the Cadenza Hub day care service also has profound implications on mitigating the underlying social inequalities in community care needs. Our study finding asserted that when appropriate policy support is in place, a pro-rich and luxurious service due to its private and self-financed nature could be transformed into an equitable primary care service model. Specifically, the Cadenza Hub day care service helps improve health equity when the services promoting healthy ageing in place work in synergy with the launch of CCSV as a graded subsidy scheme, which makes quality person-centred community care affordable to all. In contrast with many existing local social welfare schemes for low-income families that impose stringent and rigid eligibility criteria [31], the graded co-payment arrangement of the CCSV scheme is indeed in line with the approach of “proportionate universalism” proposed by Prof. Sir Michael Marmot in his first Strategic Review of Health Inequalities in England [32]. On one hand, the scale of CCSV subsidy is proportionate to the level of socioeconomic disadvantage in terms of household income; on the other hand, the wealthiest group is not left behind but still entitled to financial support with a higher level of co-payment (i.e., 40%). Hence, such a graded financial support to quality community care services ensures that health actions are universal to all but in proportion to socioeconomic and health needs. Furthermore, the financial support by CCSV has a wider equity and psychosocial impact on the disadvantaged caregivers of older adult users. With the day care service, they are temporarily relieved of the caregiving burden to focus on work in the daytime, which would otherwise not have been feasible without the CCSV support. They could also have the capacity to allocate their limited financial resources on basic necessities and health investment within their own families. Altogether, with the CCSV support, the disadvantaged caregivers could be lifted out of poverty or impoverishment due to caregiving and related health expenses, and thus stand a better chance of breaking the vicious cycle of poverty and ill-health [31].

Nonetheless, one potential barrier to achieving health equity lies in the accessibility to information regarding the existing community care services and social welfare schemes. Older adults with dementia and their caregivers tend to get frustrated when searching for appropriate services and support on their own. This issue could be improved by a better integration and promotion of the diverse existing community care services, possibly disseminated via public outpatient clinics, government-led community elderly centres across districts, relevant non-government organizations for older adults, and housing estates. Further collaboration with community providers to offer digital training and support could also enable older adults with dementia and their caregivers to access online information and recognise the tools, products, services, and activities they need, so that they can take a more proactive approach to managing their health condition with less reliance on the healthcare system [33].

### Public health implications

The public-private partnership approach to providing medico-social integration care in the community (i.e., private day care services of the Cadenza Hub supported by the publicly funded CCSV scheme in this study) is not only applicable to older adults with dementia but could be adopted more broadly as a community service delivery and financing model to address other health issues and related inequalities. To better fill the service gap beyond expanding the capacity of services in the public sector, the government could also provide financial support to users and engage community service providers (e.g., private organizations, non-government organizations, and social enterprises) which have a greater flexibility to design innovative health and social services that effectively meet the community care needs of their target audience [34, 35]. In addition, the public-private partnership is conducive to a stronger financial sustainability of community service providers by supporting part of their operational cost on human resources, technologies, and services [35]. Additional incentives such as rent waiver may also further alleviate the cost constraints; thereby promoting a better quality of community care services.

### Limitations

There are several caveats in this qualitative study. First, the relatively small number of purposefully sampled participants limited the generalizability of our findings. Caregivers who have a stronger opinion about the day care service were more likely to participate in our study, possibly leading to voluntary response bias. Second, we assessed the health impact of the day care service on older adult users with dementia based on the observations and perception by their caregivers, which may not fully reflect the first-hand experience and perceived impact of the service users. Third, the duration of day care service utilization varied significantly across users; new users and caregivers may have limited experience and thus focused only on the short-term impact of the service. Last, while the potential health equity impact of the medio-social integrated day care service with the CCSV support has been illustrated in this study, further quantitative research is warranted to provide empirical evidence on whether such as service model could effectively mitigate the existing social inequalities in community care needs in Hong Kong.

## Conclusion

The medico-social integrated day care service of the Cadenza Hub, coupled with the financial support by the government which followed the principle of proportionate universalism, appeared to have not only facilitated the improvement on physical condition, cognitive functioning, and social skills in older adults with dementia and alleviated the distress and burden of their caregivers, but also mitigated the underlying social inequalities in community care needs by ensuring need-based access to the person-centred services across the social ladder. Such a public-private partnership approach to providing medico-social integration care in the community has a potential, as a community service delivery model, to address other health issues and related inequalities.

## Data Availability

The datasets used and/or analyzed during the current study are available from the corresponding author on reasonable request.
